# The impact of restorative material and ceramic thickness on CAD\CAM endocrowns

**DOI:** 10.4317/jced.56002

**Published:** 2019-11-01

**Authors:** João-Paulo-Mendes Tribst, Amanda-Maria-de Oliveira Dal Piva, Camila-Ferreira-Leite Madruga, Marcia-Carneiro Valera, Eduardo Bresciani, Marco-Antonio Bottino, Renata-Marques de Melo

**Affiliations:** 1 DDs, MSc, PhD Student in Prosthodontics, Department of Dental Materials and Proshodontics, São Paulo State University (Unesp), Institute of Science and Technology, São José dos Campos / SP, Brazil; 2 Department of Dental Materials and Prosthodontics, São Paulo State University (Unesp), Institute of Science and Technology, Av. Eng. Francisco José Longo, n° 777, Jardim São Dimas, 12245-000 São José dos Campos, SP, Brazil; 3 Department of Restorative Dentistry, São Paulo State University (Unesp), Institute of Science and Technology, Av. Eng. Francisco José Longo, n° 777, Jardim São Dimas, 12245-000 São José dos Campos, SP, Brazil; 4 Department of Restorative Dentistry, São Paulo State University (Unesp), Institute of Science and Technology, Av. Eng. Francisco José Longo, n° 777, Jardim São Dimas, 12245-000 São José dos Campos, SP, Brazil; 5 DDs, MSc, PhD, Professor, Department of Dental Materials and Proshodontics, São Paulo State University (Unesp), Institute of Science and Technology, São José dos Campos / SP, Brazil; 6 DDs, MSc, PhD, Researcher III, Department of Dental Materials and Proshodontics, São Paulo State University (Unesp), Institute of Science and Technology, São José dos Campos / SP, Brazil

## Abstract

**Background:**

Endocrown restorations as a conservative approach to restore endodontically treated teeth still need *in vitro* investigation under fatigue and made in different materials. This study evaluated the effect of restorative material and restoration thickness on the maximum fracture load of endocrowns subjected to cyclic loading.

**Material and Methods:**

Sixty (60) third molar teeth received an endocrown preparation with three different heights of remaining dental tissue (1.5, 3.0 or 4.5 mm). A leucite-based ceramic (LEU) and a lithium disilicate (LD) based ceramic were selected to manufacture the CAD/CAM endocrown restorations, totaling 6 groups (n=10). The specimens were subjected to fatigue loading (200N, 2 x 106 cycles, water) and then to the single load to failure test (1 mm/min crosshead speed). Data were analyzed by using two-way ANOVA and Tukey tests (*p*< 0.05).

**Results:**

All endocrowns survived the fatigue test. The thickness did not influence the restoration’s fracture load (*p*=0.548) instead the restorative material (*p*=0.003). LD showed higher mean values (1714.43 N)A than LEU (1313.47 N)B.

**Conclusions:**

Endocrowns manufactured with CAD/CAM lithium disilicate blocks showed superior fracture load than the leucite-based blocks after mechanical fatigue. Nevertheless, both materials presented acceptable survival and fracture load as long as the material’s minimum thickness and the enamel adhesion are respected.

** Key words:**Endocrown, CAD/CAM, Endodontically treated teeth, Failure load, Minimal intervention dentistry.

## Introduction

Dental ceramics must have characteristics that allow them to survive successfully and work in the oral cavity (1). With the development of computer assisted design and manufacturing technology (CAD/CAM), ceramic dental systems have shown esthetic evolution and acceptable adaptation (2). However, some clinical failures in the posterior region are still commonly reported (1,3,4).

Ceramic materials’ success is directly related to an adequate adhesion dependence (5,6), preferably in enamel and distant from the cervical margin to facilitate hygiene (3,4,7,8,9), and also an adequate finishing technique to avoid biofilm accumulation (10,11). All these characteristics are not easily obtained during a coronary preparation for full crowns due to the requirements of retention, strength, structural and esthetic durability (1,12,13). Thus, other restorative treatment possibilities have become increasingly popular because they aim to preserve the dental structure, relying on enamel adhesion for the prosthetic part fixation.

Among the rehabilitative treatments proposed in the literature for the endodontically treated teeth, endocrown restorations seems quite promising (3,4,8,9,14-17). This type of indirect restoration is indicated for teeth in need of occlusion rehabilitation. Its main feature is the use of the pulp chamber as an aid in mechanical retention, dismissing the core build-ups and root canal posts; therefore being faster, cheaper and easier to manufacture (15-19). Although the internal adaptation of these restorations is not ideal (8), its longevity reaches periods of more than 5 years, and caries recurrence is the main cause of failure (20). Thus, they present a more favorable fracture pattern restoration than conventional ones (15), generally making it possible to mill a new restoration if necessary (21).

There are reports of the thermal aging effect (8,21), the restorative material type (17-19), the restored tooth anatomy (3,20,22), the fatigue limit (4) and the direction of masticatory loads application (15) on endocrown mechanical behavior. However, the papers referenced so far suggest that endocrown restoration should be applied over a thickness limit of 1.5 mm of remaining enamel. If endocrowns have this limitation, should the teeth with higher coronary portion be prepared for a full crown restoration or can they receive thinner restorations and still achieve the same results with greater tooth preservation? A theoretical study suggested that the dental tissue remnant must always be preserved (17). Moreover, when limited tooth remnant is available, even a thin restoration can protect the adhesive interface from possible adhesive failures due the stress concentration (17). Along with this question, could the use of CAD/CAM glass-ceramics with no need for further crystallization process be indicated, making the process even faster? The goal of this study was to investigate the effect of restorative material and restoration thickness on the maximum fracture load of endocrowns subjected to cyclic loading in a simulated oral environment. Thus, the null hypothesis was that the endocrown thickness and material would not negatively influence the mechanical fatigue survival and the mean values of maximum fracture load.

## Material and Methods

-Specimen preparation 

This study was approved by the local research ethics committee under the review protocol approval n° 060259/2017. A total of sixty (60) caries-free mandibular third molar teeth extracted due to periodontal disease were sampled in this study. Only teeth with discrepant sizes, with caries or restorations were excluded from the study. Next, the teeth were randomly divided into six groups according to the factors: “restorative material” and “restoration thickness” ( Table 1).

**Table 1:**
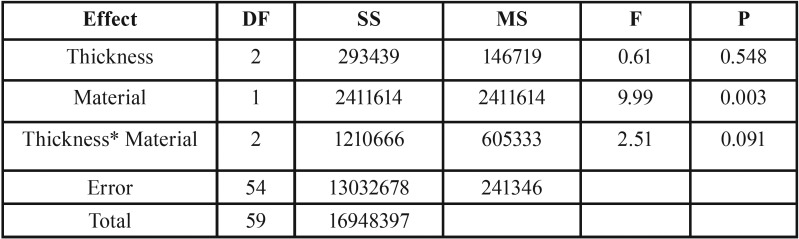
Results of two-way analysis of variance and the interaction terms for load to fracture mean values, according to the endocrown thickness and material (*p*< 0.05).

The teeth were sectioned according to the restoration thickness factor. Thus, teeth from subgroups 1.5, 3.0, and 4.5 mm were sectioned at 4.5, 3, and 1.5 mm from cement-enamel junction, respectively. The cut was made with a diamond disc (7070; KG Sorensen) under constant water irrigation. Access to the pulp chamber was accomplished by using a high-speed handpiece (MRS-400 PB; Dabi Atlant) and a diamond bur (3131; KG Sorensen) with copious water spray. Pulpal remnants were removed and root instrumentation was performed with hand files (Kerr). The teeth were prepared by the same operator under constant water with a specific device to standardize the preparations in 4 mm width and thickness. The height was standardized according to the pulp chamber floor flattened with resin cement (Variolink II; Ivoclar Vivadent). Then, the teeth had their cervical margin prepared by using a wheel bur held parallel to the occlusal plane (3153; KG Sorensen) and the coronal pulp chamber and endodontic access cavity continuously with using a cylindrical-conical diamond bur (2136; KG Sorensen) (23). The prepared teeth were embedded into an acrylic resin approximately 1.00 ± 0.15 mm below the cement-enamel junction.

**Figure 1:**
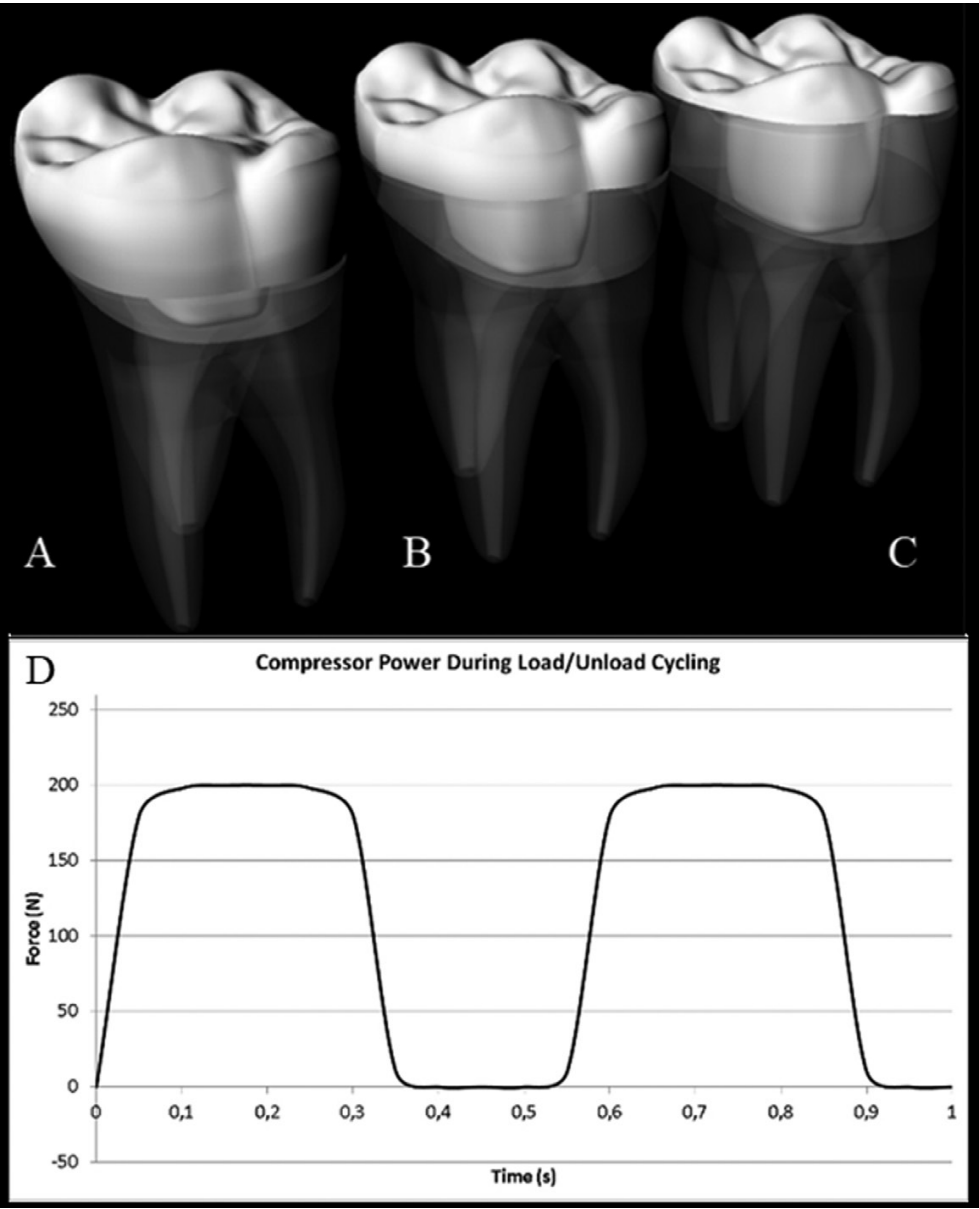
Restoration minimum thickness and tooth remnant height. Subgroup 4.5 (A), 3.0 (B) and 1.5(C) with remnant tooth support of 1.5, 3.0 and 4.5 mm high, respectively. Fatigue test profile during mechanical cycling (D).

Next, each tooth was scanned (PlanScan; Planmeca) and the endocrowns restorations were generated in a CAD system (Romexis 4.5.0.R; Planmeca) with the requirements for full anatomic restorations of molar teeth. The materials used to design and manufacture endocrown restorations were leucite-based IPS Empress CAD (Ivoclar Vivadent AG) and lithium disilicate-based IPS e.max CAD (Ivoclar Vivadent AG). The difference between the groups followed a ratio of 1.5 mm in the three chosen restoration thicknesses (Fig. 1). Restoration milling was performed under constant water cooling. After being milled, following Villefort *et al.* (2017) protocol (24), the restorations were separated from the block-holder, evaluated by stereomicroscopy (Discovery V20; Carl Zeiss) and then, cleaned with distilled water (Vitasonic II, VITA Zahnfabrik) for 10 minutes and left to dry. Lithium disilicate crystallization process followed the manufacturer’s protocol in a specific oven (Programat P700; Ivoclar Vivadent) (25).

The total-etch adhesive technique was applied following the visual accuracy check for each restoration: the groups of lithium disilicate-based ceramic were etched with 10% hydrofluoric acid (Condacporcelana, FGM) for 20 seconds, rinsed with water (20 seconds) and dried (6). The groups of glass-ceramic leucite-based were etched with 5% hydrofluoric acid (Fórmula e Ação Farmácia) for 60 seconds, rinsed with water (15 seconds) and dried. Then, a thin coat of silane agent (Monobond N; Ivoclar Vivadent) was applied in all restorations with a microbrush and it was allowed to react for 60 seconds.

The tooth preparation was treated with phosphoric acid (37% CondAC-37; FGM), rinsed with water, and dried. Then, a layer of bond agent (Excite F DSC; Ivoclar Vivadent) was applied for 10 seconds and dried.

A dual-cured resin cement (Variolink N; Ivoclar Vivadent) was poured and mixed (1:1) and applied to the intaglio surfaces of the endocrowns. Each restoration was seated on the respective tooth preparation and then held in position by a metal rod (750 g, 5 minutes) positioned in the occlusal surface (24). Excess cement was gently removed after 2 s with initial light activation, and each specimen was light-activated for 20 seconds on each surfaces (High Power program, Bluphase N; Ivoclar Vivadent). The specimens were stored (distilled water at 37°C) for 24 hours prior to fatigue test.

-Fatigue test 

**Figure 2:**
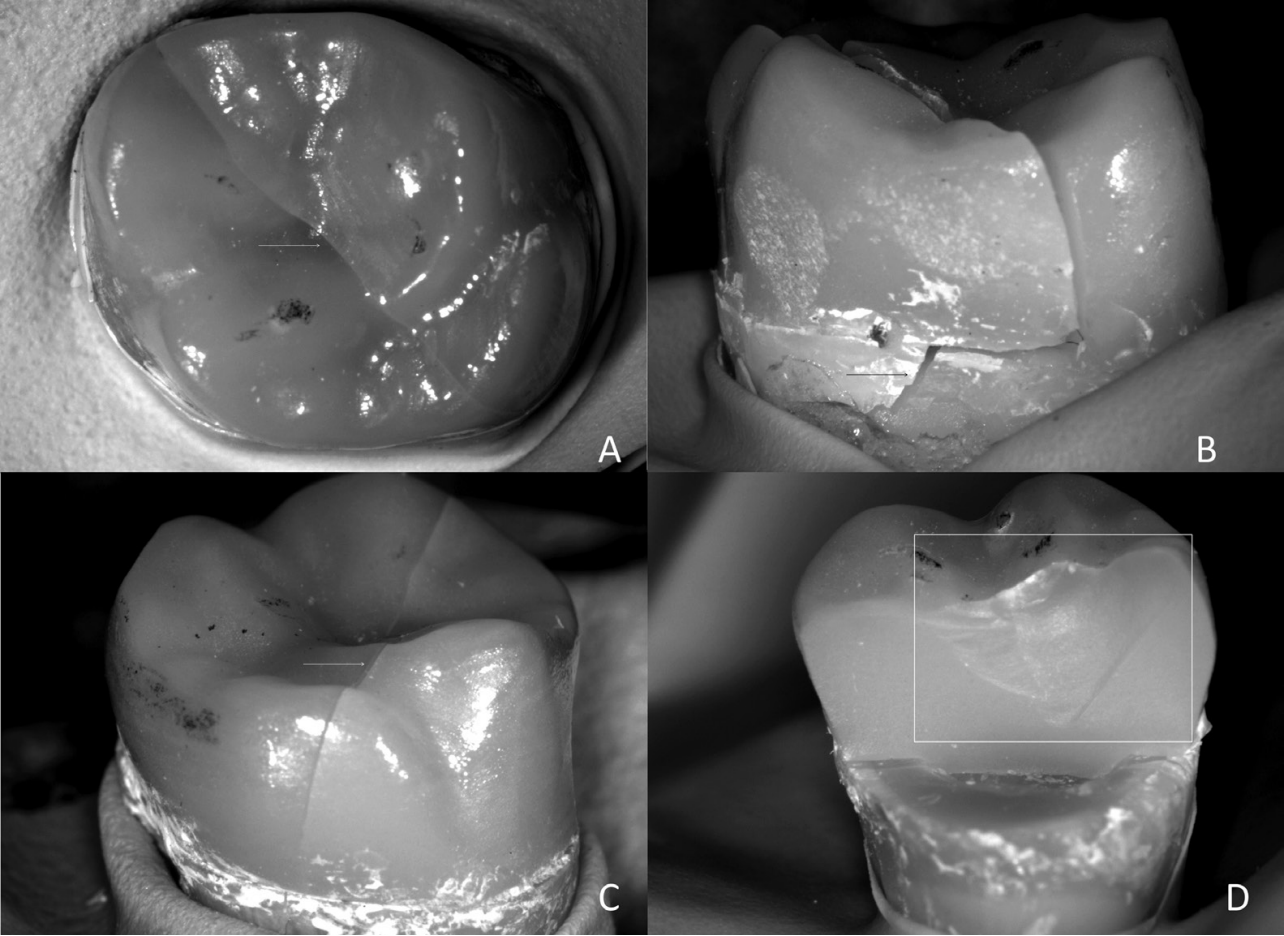
Endocrowns failure analysis view assessment revealing (A-C) the bulk failure profile separating the crown into two main pieces (white arrows) even involving dental fracture (black arrow), and (D) an overview (stereomicroscopy) of fractographic marks suggesting the crack propagation from the occlusal surface.

The fatigue test was set for 2 x 106 cycles, 26 at 2 Hz frequency under water at 37°C (Fig. 2). All specimens were loaded vertically on the occlusal surfaces (tripoidism) with 200 N. The loading was applied by using a round stainless steel piston with 6 mm diameter (24). The specimens were checked for cracks, chipping, or fracture in every 100,000 cycles after each loading phase through the transillumination technique (27). Failure was defined as large chippings, cracks, or ceramic bulk fracture.

-Fracture load test

After the suspension of the fatigue test with 100% survival rate, all specimens were subjected to a single load to failure test in a universal testing machine (DL-1000; EMIC; 1 mm/min crosshead speed, 1000 kgf load cell). The specimen was fixed in the horizontal plane with no inclination, and the load was applied vertically until failure by using the same indenter used in the fatigue loading. This test was performed to evaluate the maximum load to fracture (in N) and detect any difference between the groups.

-Fractography analysis

**Figure 3:**
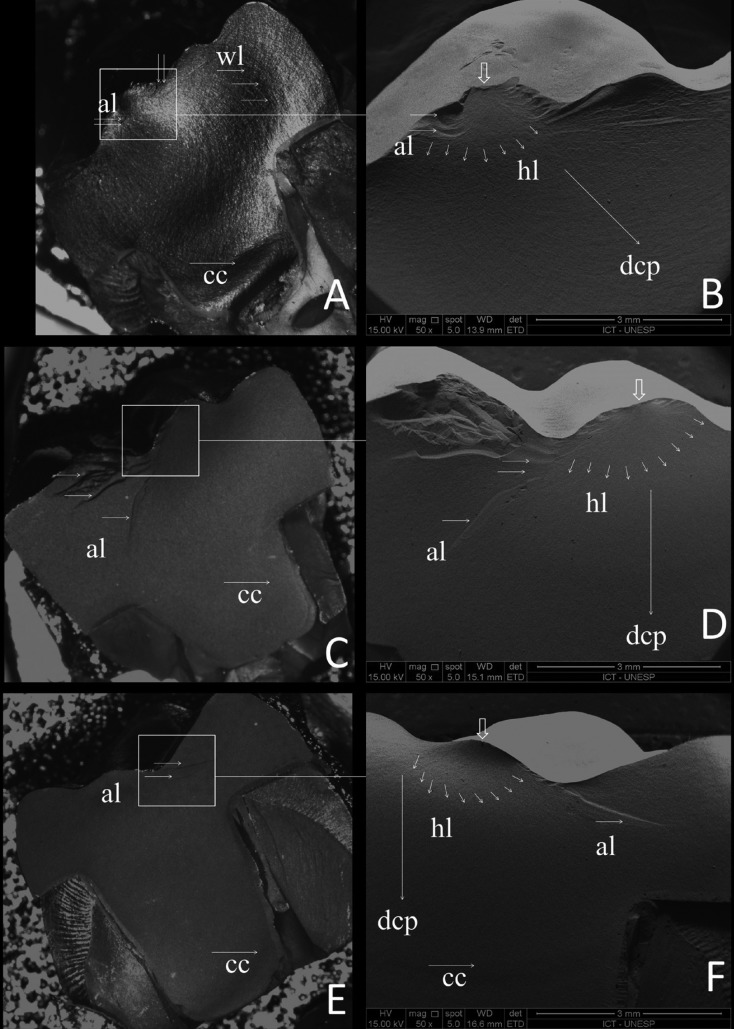
Fractography analysis of the endocrown restorations. (A, C and E) Stereomicroscope pictures (25x) of the reassembled fractured specimen. SEM (50x) micrographies (B, D and F) shows the loading damage detected on the occlusal surfaces of the restoration where the failure origin was located. The white squares show the origin of the fracture at the contact points. Hackel lines (hl), walner lines (wl) and arrest lines (al) are indicating the direction of crack propagation (dcp) and the compression curl (cc) was observed at the bottom of the restoration.

Each fractured crown was visually inspected at 25x magnification (Zeiss Discovery V20; LLC) (Fig. 3). One representative sample of each group was inspected by using a scanning electron microscope (Inspect S50; FEI). The specimens were sputter coated with gold for 180 s at 40mA, creating a 30nm-thick layer. This was examined under different standard SEM magnifications operated at 20KV with secondary electron detection by a single operator.

-Data analysis

A descriptive statistical analysis of fracture load values (mean and standard deviation) was performed. Differences between the groups were analyzed by two-way analysis of variance (ANOVA) followed by Tukey multiple comparison test with the level of significance set at 95% by using Minitab statistical software (Minitab, Version 14.12, 2004). The sample power of 92.95% was obtained with an open source calculator (www.openepi.com) with 95% two-tailed confidence interval.

## Results

100% of the specimens survived the fatigue loading with no signs of cracks or chipping. All crowns had their maximum load to fracture detected. Two-way ANOVA reveled that load to fracture was only significantly affected by the material ( Table 1).  Table 2 shows that lithium disilicate significantly increased the fracture load of the endocrowns compared with groups manufactured with leucite-based ceramic (*p*=0.003). Thus, lithium disilicateA performed better than leuciteB. The fracture loading of teeth restored with endocrowns did not differ statistically in regard to the different occlusal thicknesses used (*p*=0.548).

**Table 2:**
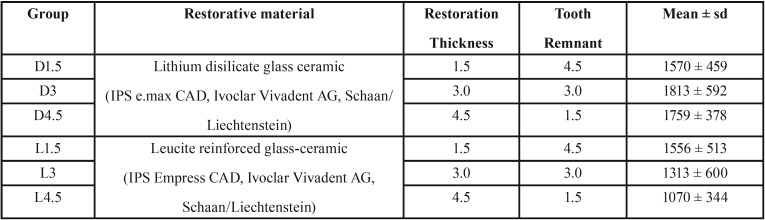
Single load to failure mean values (N) and standard deviation (sd) of all test groups.

According to failure analysis, the crowns predominantly failed by bulk fracture. Two fracture modes with different numbers of main fractured pieces were observed. Fractures extended along the mesio-distal plane occurred in 85% of the specimens, separating the crown into two main pieces. Fractures extended further to the lingual groove in the 15% of the crowns, resulting in three main fractured pieces (Fig. 3). Representative failure samples were then submitted to scanning electron microscopy (SEM) for further investigation of failure origin. Fractographic analysis revealed that the fracture origin was always at the occlusal surface in all fractured restorations, mainly from the major contact loading area, underneath the indenter and corono-apically propagated.

## Discussion

The goal of this study was to investigate the effect of restorative material and restoration thickness on the maximum fracture load of endocrowns subjected to cyclic loading. The results suggest that the restorations thickness does not influence the survival and maximum fracture load of the evaluated endocrowns. However, the use of lithium disilicate-based ceramics showed superior mechanical results than the glass-ceramics, partially accepting the study hypothesis.

The impact of the restoration thickness on the maximum fracture load was previously investigated for full crowns (1,12,13,28). Several papers have used simplified geometry models due to the complexity of the mechanical principles (13,29,30). However, an anatomical model is close to the geometric characteristics of the indirect restoration and may represent a more accurate specimen in *in vitro* studies (1). Although a monotonic test results in overestimated mechanical strength values, it can be applied to rank the evaluated materials (31). However, fatigue tests where the test specimens are subjected to stress accumulation in a susceptible environment to generating a crack tend to present lower strength values than those obtained by means of monotonic tests (8,32,33). In this study, all restorations were submitted to a mechanical fatigue test to investigate the influence of restorative material thickness and type on the restoration’s survival. With unanimous survival between groups, it was possible to make an inference between number of cycles and usage time in years, suggesting that these restorations would have a lifetime of more than 4 years (26). The 200 N load was selected in this study because it consists of a mean value existing in the posterior region of a patient without parafunctional habits (26,34). In addition, the tripoidism contact was adopted to approximate the physiological load application where more than one contact point is present in the lower molars (24,35).

According to the manufacturer’s instructions, the recommended ceramic thickness on the occlusal surface is 1.5 mm, being assumed herein as the experiment’s minimum thickness. However, endocrown fabrication is not indicated for glass-ceramics, even if this minimum thickness is maintained (36). However, some studies have evaluated endocrown restorations made of feldspathic ceramics, and it was detected that there are no such high risks in their indication for this treatment (8,9). In finite element numerical simulations, some authors defend the use of glass-ceramics as the choice material for endocrown production (18,19). Another fact that suggests acceptable behavior of feldspathic ceramics is the report of its use for thin crowns of 1.5mm thick with adequate survival (13). Glass-ceramic use would bring the esthetics closer to natural teeth, as well as reduce the laboratory cost because the crystallization process is not required when compared to lithium disilicate (36). Also, leucite was suggested as a promising alternative to lithium disilicate for manufacturing endocrown restorations due to its better stress distribution (17).

Despite the same result under mechanical fatigue, the lithium disilicate showed superior mechanical behavior in requiring higher load to fracture. Lithium disilicate is indicated for endocrown restorations as long as the enamel adhesion is maintained and the minimum material thickness of 1.5 mm is respected according to the manufacturer. The preeminence of this ceramic’s average load to fracture is explained by the disilicate crystals’ arrangement, hindering crack propagation and mechanical failures (37) in comparison with the leucite used in this study.

Both ceramics have a glass matrix and are subjected to surface treatments in order to produce promising bond strength results (6,38,39). According to the manufacturer, this material’s exposure to hydrofluoric acid occurs at different times to meet the individual conditions of each structure. Since acid conditioning acts on the glass matrix, a longer exposure time (60 s) is required in leucite due to the greater amount of glass matrix on the surface compared to restorations made from lithium disilicate (6). This difference is needed to avoid disilicate crystal overexposure or superficial defects capable of initiating premature fracture of the material (40). Thus, adequate adhesion was achieved and there were no cases of any restoration detachment.

The studies that analyzed endocrowns kept a minimum enamel thickness of 1.5 mm (3,4,19,21,22,41). Herein, three different restoration heights within the range established by minimum enamel thickness (1.5 mm) and minimum restorative material thickness (1.5 mm) were evaluated. This factor was not significant for the results, suggesting that it is possible to restore a tooth with a ceramic endocrown without preparing an extensive axial walls. Clinically, this finding allows us to assume that a tooth that is endodontically treated and has a vertical occlusion dimension reduced by wear, fracture, caries or other reason does not need to receive a composite filling, a full crown associated to a fiber post or an extensive endocrown. Simple axial wall flattening in the cervical direction and the preparation of expulsive walls in the pulp chamber would make it possible to use a thin endocrown to safely restore this molar (17).

Some studies have evaluated endocrown preparations with a ferrule (22,42-44). However, if the endocrown cementation is adhesive, reducing an amount of sound enamel represents an inappropriate situation since the adhesion between ceramic and tooth promoted by the enamel is superior in comparison to the dentin (45). Moreover, these studies have observed that the ferrule causes a greater number of defects in the tooth due to an existing lever in the dental root (42,44).

Some *in vitro* studies demonstrate and classify defects in endocrowns as restorative adhesive failures (4,22). However, adhesive failure is not commonly clinically reported (20). The adhesive failure was quoted only twice; one of which was due to the restored tooth being served as a removable prosthesis support (16). In the current study, the restorations presented 0 failure chances after 2 million mechanical fatigue cycles with 200N load. Longitudinal studies demonstrate a survival rate of more than 95% of the endocrowns, not being higher due to caries recurrence (20), periodontal disease, endocrown debonding, minor chipping and major fractures (16). This high survival rate can be explained by the prevalence of axial loads on molars. Nevertheless, the clinical success of endocrowns in premolars is not as high due to oblique forces (15,46).

All tested specimens failed catastrophically after a single load to failure test. This fracture type propagating throughout the restoration is reported as a common failure in *in vivo* (47,48) and *in vitro* studies (1,41,49). The fracture failure mode is directly related to the ease of crack propagation inside the ceramic material (50). In this way, no repairable fractures were observed since all constituted a great part of the restoration. However, the load values needed to fracture the restoration were higher than the maximum bite force mean values (284.9 N for men and 304.9 N for women) (43,51), suggesting that both materials can be indicated for endocrown manufacture regardless of the thicknesses.

Although a periodontal ligament layer was not simulated, some studies have reported that this is not necessary (1,52). Other studies made representative anatomical teeth specimens lacking this dental tissue (3,26,41). This is preserved so that the damping effect does not soften the cyclic fatigue in restorative materials.

According to the fractographic analysis, the fractures respected a failure pattern due to the load application. The failure origin occurred on the restoration’s external surface in the indenter contact region. Hackle lines are identified in the SEM images and indicate the crack propagation direction from the top to bottom. Under this study’s limitations, lower molars restored with the endocrown modality by using different materials and thicknesses showed greater fracture strength. However, these results may not be clinically significant since the failure load data are higher than the normal mean values of masticatory load (53). Therefore, future clinical investigations should be developed to investigate the *in vivo* performance of endocrowns.

## Conclusions

From this study, the following were drawn:

Endocrowns manufactured with CAD/CAM lithium disilicate blocks showed superior fracture load than the leucite-based blocks after mechanical fatigue. Nevertheless, both materials presented acceptable survival and fracture load as long as the material’s minimum thickness and the enamel adhesion are respected.
